# Ghrelin-Mediated Regeneration and Plasticity After Nervous System Injury

**DOI:** 10.3389/fcell.2021.595914

**Published:** 2021-03-25

**Authors:** Irina Stoyanova, David Lutz

**Affiliations:** ^1^Department of Anatomy and Cell Biology, Medical University Varna, Varna, Bulgaria; ^2^Department of Neuroanatomy and Molecular Brain Research, Ruhr University Bochum, Bochum, Germany

**Keywords:** GHSR, ischemia, stroke, brain and spinal cord injury, synaptic activity, neurogenesis

## Abstract

The nervous system is highly vulnerable to different factors which may cause injury followed by an acute or chronic neurodegeneration. Injury involves a loss of extracellular matrix integrity, neuronal circuitry disintegration, and impairment of synaptic activity and plasticity. Application of pleiotropic molecules initiating extracellular matrix reorganization and stimulating neuronal plasticity could prevent propagation of the degeneration into the tissue surrounding the injury. To find an omnipotent therapeutic molecule, however, seems to be a fairly ambitious task, given the complex demands of the regenerating nervous system that need to be fulfilled. Among the vast number of candidates examined so far, the neuropeptide and hormone ghrelin holds within a very promising therapeutic potential with its ability to cross the blood-brain barrier, to balance metabolic processes, and to stimulate neurorepair and neuroactivity. Compared with its well-established systemic effects in treatment of metabolism-related disorders, the therapeutic potential of ghrelin on neuroregeneration upon injury has received lesser appreciation though. Here, we discuss emerging concepts of ghrelin as an omnipotent player unleashing developmentally related molecular cues and morphogenic cascades, which could attenuate and/or counteract acute and chronic neurodegeneration.

## Introduction

Nervous system injury is a remarkably complex process which includes blood-brain-barrier interruption, vascular damage, severe consequences of unsustainable hemorrhage and aberrant metabolic state. The intricacy of damage is due to activation of neuronal and glial apoptosis, release of molecules suppressing regenerative responses and attraction of immune cells as well as microglia activation. Therefore, it might seem peculiar to expect that a single molecule could provide effective treatment of this complex trauma, let alone entail a complete functional recovery. However, a growing body of evidence suggests that the complexity of injury can be counteracted with pleiotropic molecules able to stimulate manifold regenerative events. In addition to multifaceted functions, a good candidate for treatment of nervous system injury must give robust and reproducible effects in different experimental models of injury (Kessler, 2010). Of note, the time frame of therapeutic intervention after injury is critical (Lee et al., 2010; le Feber et al., 2016) and no single intervention with a candidate of interest can be expected to easily address all demands of the regenerating nervous system (Kessler, 2010).

Experimental evidence collected over the past two decades has raised the possibility that the 28 amino acid-long and evolutionary conserved neuropeptide hormone ghrelin may have a therapeutic potential for treatment of nervous system injury. In this review, we have focused on different schools of thought and heuristics related to ghrelin’s roles in nervous system repair and discuss the emerging concepts of ghrelin-mediated regeneration.

## Ghrelin Expression and Signaling

### Ghrelin Regulates a Multitude of Physiological Processes in the Body

Increasing body of evidence suggests that the gastric hormone ghrelin may have various physiological functions in addition to its primary role to activate the hypothalamic orexigenic neurons (Al Massadi et al., 2017). Initially, ghrelin has been found in the enteroendocrine cells of the gastric fundus (Kojima et al., 1999; Sakata et al., 2002), followed by reports on its expression in other organs, such as kidney (Mori et al., 2000), placenta (Gualillo et al., 2001; Allbrand et al., 2018), Leydig and Sertoli cells of the testis (Barreiro et al., 2002), pancreas (Date et al., 2002; Wierup et al., 2002), and distinct brain areas (Kojima et al., 1999; Cowley et al., 2003; Hou et al., 2006; Cabral et al., 2013; for further review see also Stoyanova, 2014). However, these data are quite inconsistent, probably due to methodological differences. As discussed in detail by a recent review, ghrelin is even not synthesized at physiologically relevant levels in the mammalian brain (Cabral et al., 2017).

The ghrelin gene encodes pre-proghrelin, which is cleaved to proghrelin. Proghrelin is further cleaved to produce unacylated ghrelin (desacylghrelin or DAG), devoid of endocrine functions but able to suppress cell proliferation (Cassoni et al., 2001; Broglio et al., 2003), and an acylated form referred to as ghrelin (Hosoda et al., 2000): its highly conserved N-terminal backbone is post-translationally acylated (Kojima et al., 1999; Ariyasu et al., 2001) by the enzyme ghrelin-O-acyl transferase (GOAT, Yang et al., 2008). Esterification of approximately 20% of the circulating ghrelin in humans takes place in the ghrelin-secreting cells, where the N-terminus of the molecule is hydrophobically tailored (Hosoda et al., 2000). It is very likely that there is a hydrophobic interaction between the n-octanoyl group and the growth hormone secretagogue receptor (GHSR1a), which allows recognition and binding of ghrelin (Bednarek et al., 2000).

Since GOAT is required for the synthesis of ghrelin it is interesting to analyze whether the distribution of GOAT expression may match with the potential places of ghrelin production. In humans, GOAT mRNA transcripts are expressed mainly in the stomach and pancreas (Gutierrez et al., 2008), and in the vast majority of organs according to Lim and collaborators (Lim et al., 2011). Such transcripts have been detected in the murine intestines, testis, pituitary gland, and hypothalamus (Sakata et al., 2009), and at very low levels in the porcine brain, liver, lungs, ovaries, and muscles (Lin et al., 2011). Co-expression of GOAT and ghrelin has been found in the stomach but fewer of the duodenal ghrelin-positive cells had contained GOAT, which could be explained with the morphological differences between the cells: only the so-called closed-type ghrelin cells express the enzyme (Sakata et al., 2009). Another research group has considered that the levels of ghrelin- and GOAT-gene expression are quite low, thus challenging the proper mapping of ghrelin’s secretion as well as the clarification of whether hypothalamic pre-proghrelin mRNA is translated to protein and whether both pre-proghrelin and GOAT mRNA are present in the same cells (Cabral et al., 2017).

There are hypotheses suggesting that ghrelin is degraded or modified in tissues and blood by other unknown enzymes (De Vriese et al., 2004; Satou et al., 2011) which might affect ghrelin’s structure (post-translational modifications, truncation) and functions (interaction with GHSR1a and other partners, ability to cross the blood-brain barrier). It seems that variations in the protein backbone as well as post-translational modifications orchestrate the ability of ghrelin to cross the blood-brain barrier and its retention within the brain tissue (Banks et al., 2002). The brain accessibility of ghrelin has been thoroughly reviewed by Perello and colleagues (Perello et al., 2019). Furthermore, there is little consistency in the general view on the relationship between structure and functions of ghrelin. Early studies have demonstrated that the first 4–5 residues of ghrelin are sufficient to functionally activate GHSR1a to the same extent as the full-length peptide does (Bednarek et al., 2000; Satou et al., 2011). Yet, according to Torsello and collaborators, the molecular length is not crucial for ghrelin’s activity (Torsello et al., 2002). These contradictory findings raise researchers’ awareness of the significance of ghrelin’s truncation and demand additional experimental evidence.

Ghrelin circulates in the blood stream (Date et al., 2000) and in the cerebrospinal fluid (Mogi et al., 2004). Though it seems difficult to determine the amount of circulating DAG in healthy human plasma under optimal sample handling and assaying conditions, the amount of the modified form has been estimated as 10% of the total circulating peptide (Ariyasu et al., 2001). Ghrelin’s concentration in the human plasma has been measured to be 117 ± 37.2 fmol/μl, which corresponds to 0.117 μM. Given that the molecular weight of human ghrelin is 3370.9 g/mol (according to PubChem^1^, ghrelin is also known as lenomorelin, compound CID: 91668172), the mass concentration of ghrelin in the plasma can be calculated as 394.4 ng/l. For a sample of 15 μl volume which shall be subjected to immunoblotting, the concentration of ghrelin will be 0.006 ng, which appears to be far below the detection limits of the method (approximately 0.1 ng of protein for an average standard immunoblot analysis). Furthermore, in the presence of serine proteases and HCl no accurate measurements are possible because they lead to de-acylation of ghrelin (Blatnik et al., 2012). Nonetheless, there are some other methods for quantitative assessment of ghrelin in plasma and tissue samples such as the reverse phase high performance liquid chromatography, radioimmunoassay (Date et al., 2000), sensitive immunoblotting (Shanado et al., 2004), and mass spectrometry combined with liquid chromatography (Rauh et al., 2007).

Interestingly, ghrelin is the only known endogenous ligand activating the GHSR1a (Kojima et al., 1999; Bednarek et al., 2000). Of note, the receptor is highly expressed in the nervous system (Guan et al., 1997; Muccioli et al., 1998; Mitchell et al., 2001; Zigman et al., 2006; Andrews, 2011; Bron et al., 2013). GHSR1a is a G protein-coupled receptor product of the *Ghsr* gene, which encrypts two variants of GHSR mRNA, type 1a and 1b (Petersenn et al., 2001). GHSR1a operates in two different activation modes: an agonist-induced, ghrelin binding and a constitutive mode (Holst et al., 2003). Neither does GHSR1b bind ghrelin nor has this receptor form been known to exert a signal transduction activity (Howard et al., 1996). However, both receptor forms can heterodimerize within the endoplasmic reticulum and decrease the constitutive activity of GHRS1a by attenuating its cell surface expression (Chow et al., 2012). The agonist-induced receptor activity is involved in the control of energy balance as well as in hedonic and addictive aspects of eating (Perello et al., 2010; King et al., 2011; Perello and Dickson, 2015). The constitutive functional mode of ghrelin’s receptor is chronic and triggered by scarce specific inverse agonists (Holst et al., 2007). It significantly reduces pre-synaptic voltage-gated calcium (Ca_V_) channel trafficking (López Soto et al., 2015) by elevating the retention of Ca_V_ complex in the endoplasmic reticulum. Because Ca_V_2.1 and Ca_V_2.2 channels are involved in calcium-induced transmitter release, their inhibition by GHSR1a’s constitutive activity could be relevant in brain areas with high density of ghrelin receptor but with limited access for ghrelin, such as the hippocampus (Cabral et al., 2015).

The tissue distribution of GHSR1a has been extensively studied by means of different neuroanatomical techniques. GHSR1a expression has been shown in distinct brain areas with varying degrees of neuronal plasticity. For example, *in situ* hybridization histochemistry has divulged particularly high levels of GHSR1a in the arcuate (ARH) and ventromedial hypothalamic nucleus (VMH) (Howard et al., 1996; Bennett et al., 1997), but the method is not sensitive enough toward cell surface receptors with relatively low mRNA levels. With novel cRNA probes the mRNA encoding the functional GHSR1a was confirmed in brain areas previously unknown to express GHSR1a (Zigman et al., 2006). Moreover, these cRNA probes allow the study of co-localization of GHSR1a with certain neurotransmitters, i.e., dopamine and cholecystokinin. Such findings indicate that ghrelin can selectively amplify dopamine signaling in neurons co-expressing dopamine-1 receptor and GHSR1a. During the last years, different mouse models have been engineered to express reporter genes driven by transcriptional regulatory regions of the gene-of-interest. Such targeted knock-in approach with replacement of GHSR1a coding region with that of β-galactosidase (Diano et al., 2006) has allowed the visualization of cells that would express GHSR1a. However, this mouse model is not suitable for simultaneous functional studies (Mani et al., 2014), whereas mouse models carrying tau green fluorescent protein (eGFP) downstream of an internal ribosome entry site have been more appropriate for such studies (Jiang et al., 2006), thereby revealing eGFP-immunoreactivity in brain areas lacking or barely expressing GHSR1a (Mani et al., 2014).

Ghrelin, GOAT, and GHSR1a form a functional triad that is evolutionally conserved (Tine et al., 2016) and triggers downstream signaling (Hou et al., 2016; Bender et al., 2019) to the cell nucleus, thus affecting gene expression underlying neuronal survival and plasticity (Dolmetsch et al., 2001). Interestingly, the majority of circulating ghrelin is DAG (Ariyasu et al., 2001), and unable to activate GHSR1a, but DAG can act independently as a hormone or together with ghrelin to modulate physiological and pathological processes (Broglio et al., 2004; Thompson et al., 2004; Pacifico et al., 2009; Delhanty et al., 2014). Moreover, intraperitoneal administration of ghrelin or DAG activates *c*-Fos in the ARH, while when simultaneously injected, DAG abolishes the effect of ghrelin on neuronal activity (Inhoff et al., 2008).

Since its discovery ghrelin has been speculated as an orexigenic molecule regulating energy homeostasis and body weight as well as an initiator of eating, because ghrelin’s levels rise before meals and fall shortly after feeding (Cummings et al., 2001; Tschöp et al., 2001; Cummings, 2006; Nass et al., 2008, 2011; Kojima and Kangawa, 2010). Mouse models with overexpression or genetic deletion of ghrelin, GHSR1a and GOAT, or deficiency of both ghrelin and GHSR1a, have been used to study the role of each of the ghrelin-triad components in eating behavior and metabolism control (Sun et al., 2003, 2004; Wortley et al., 2004; Perello et al., 2010; Zhao et al., 2010; Davis et al., 2012; McFarlane et al., 2015). Moreover, models with site-selective expression of GHSR1a provide evidence that ghrelin plays a role in stress-induced eating (Chuang et al., 2011). Genetically manipulated models have further demonstrated that ghrelin is a regulator of blood glucose levels under conditions of famine (McFarlane et al., 2014). A broadened view on ghrelin as a key player in seeking and storage of energy and also in mitigation of energy shortfall has emerged (Tschöp et al., 2000; Muller et al., 2001; Gauna et al., 2004; Delhanty and van der Lely, 2011; Andrews, 2019); for a more detailed review of these experimental set-ups and their outcome see also Uchida et al. (2013).

The pleiotropic image of ghrelin is created by the complexity of signaling pathways modulated by the peptide. On the one hand, ghrelin is a unique substrate for GOAT (Darling et al., 2015), but when circulating in the blood stream, the peptide is prone to the activity of several esterases reversing the process of acylation (De Vriese et al., 2004). When reaching a target cell with a surface-exposed GOAT, DAG can be re-acylated to activate local GHSR1a-dependent downstream signaling (De Vriese et al., 2004; Gahete et al., 2010; Hopkins et al., 2017; Murtuza and Isokawa, 2018; for a review also see Abizaid and Hougland, 2020), which induces phosphorylation of extracellular regulated protein kinases ERK1/2, phosphatidylinositol 3 kinase (PI3K), and protein kinase B (Kohno et al., 2003, 2008). These findings suggest that on-demand, a local re-acylation of DAG at the target cell surface might be enabled by retrieval of the necessary amount of DAG from the circulating repository; however, the hypothesis needs further confirmation.

It should be noted that in Parkinson’s disease (PD) patients with dementia, the ratio ghrelin/DAG is considerably lower as well as the number of GOAT-positive cells within the hippocampal granule cell layer. Therefore, less hippocampal cells may acylate DAG, which would result in reduction of ghrelin-stimulated GHSR1a signaling and cognitive deficits (Hornsby et al., 2020). These findings shed more light into the adult brain plasticity and suggest new therapeutic strategies for treatment of mnemonic dysfunction.

On the other hand, the recently discovered liver-expressed antimicrobial peptide 2 (LEAP2) has been also recognized as an endogenous ligand for GHSR1a acting in an antagonistic manner to ghrelin (Ge et al., 2018). LEAP2 fully inhibits GHSR1a activation by ghrelin and averts the major effects of ghrelin during chronic caloric restriction. Of note, the LEAP2/ghrelin ratio determines the GHSR1a-dependent signaling, whereby domination of LEAP2 blocks binding of ghrelin to GHSR1a (Mani et al., 2019). However, LEAP2 may suppress ghrelin effects via other mechanisms, independently of GHSR1a, based on an inverse relationship that has been found between the plasma levels of both proteins when nutritional status changes (Ge et al., 2018). The active part in LEAP2 inhibiting the ghrelin receptor is the N-terminal region, as determined recently by M’Kadmi and collaborators (M’Kadmi et al., 2019). LEAP2 and its N-terminal act as inverse agonists of GHSR1a by stabilizing an inactive conformation of the receptor, thus antagonizing ghrelin at triggering inositol phosphate 1 production and Ca^2+^ mobilization. Hence, the endogenous inverse agonist of GHSR1a has drawn attention to development of anti-obesity pharmacological agents. Systemic LEAP2 administration in mice suppresses only ghrelin-stimulated food intake and growth hormone release (Ge et al., 2018), while in rats intraventricularly injected LEAP2 completely abolishes the central ghrelin effects (activation of the hypothalamic neurons, promotion of food intake, increase of blood glucose level, and body temperature reduction) but does not inhibit the central effects of DAG (Islam et al., 2020). However, it is still unclear whether LEAP2 can cross the blood-brain-barrier similarly to ghrelin in order to affect GHSR1a in the central nervous system (Abizaid and Hougland, 2020). Further experiments have shown that GHSR1a can form complexes with melanocortin accessory protein 2 (MRAP-2) in the ventral hypothalamus to benefit the orexigenic effects of ghrelin (Srisai et al., 2017).

### Ghrelin Modulates Downstream Signaling Cascades in the Developing and Adult Nervous System

There is no ghrelin production in the stomach of the rat fetus until embryonic day 19 (Hayashida et al., 2002), yet the fetal plasma levels of DAG are 5- to 10-fold higher than this in the maternal circulation (Nakahara et al., 2006). The maternal peptide is transferred to the fetal circulation during the second half of the pregnancy (Nakahara et al., 2006) and DAG in the amniotic fluid has been shown to stimulate fetal skin and spinal cord development (Sato et al., 2006). The maternal-fetal ghrelin traffic appears to be neuroprotective and important for neurogenesis, as exogenous chronic treatment of the mother with ghrelin can prevent neural tube defects in the fetus (Yuzuriha et al., 2007).

Ghrelin and DAG have differential effects on neurogenesis in distinct parts of the brain in a time-dependent manner. They promote neurogenesis including proliferation of neuronal precursor/stem cells during fetal development via GHSR1a and another yet undefined way for DAG (Nakahara et al., 2006; Sato et al., 2006) which seems to become unfunctional in precursor cells after parturition. Immunohistochemistry in combination with GHSR-eGFP reporter mice has more precisely revealed GHSR1a in several brain regions, including the olfactory bulb (OB) but not in the subventricular zone (SVZ) of the lateral ventricle (Ratcliff et al., 2019). Surprisingly, ghrelin seems to have no direct effect on the neuronal stem/precursor cells in the SVZ and does not increase adult OB neurogenesis. In contrast, fasting and re-feeding activate newly formed OB cells in ghrelin-mediated manner, suggesting that these newborn cells are uniquely sensitive to changes in ghrelin levels (Ratcliff et al., 2019). In the hippocampal neurogenic niche, GHSR1a is expressed in mature granule cells of the dentate gyrus and elevated ghrelin levels inflicted by fasting seem to enhance adult neurogenesis and memory (Kent et al., 2015; Hornsby et al., 2016).

The hypothalamic arcuate nucleus (ARH) is a key point of the neuronal network regulating body weight and energy homeostasis. The ARH neurons are directly regulated by leptin and ghrelin (Dickson, 2002; Elmquist et al., 2005). These neurons project to the paraventricular nucleus (PVN) and are considered as a major regulator of food intake (Williams and Elmquist, 2012). The ARH neurons can undergo GHSR1a-mediated morphological and functional remodeling under energy deprivation (Cabral et al., 2020). This makes the ARH nucleus very useful for studying the effects of ghrelin on neuroplasticity under such conditions. Ghrelin’s activity during the perinatal period is important not only for proper maturation of axonal projections of the ARH neurons but also affects the adult metabolic regulation. Thereby, correct timing and magnitude of ghrelin’s action are crucial, because both excess as well as shortage of ghrelin at that period cause alterations in hypothalamic development and long-term metabolic perturbations (Steculorum et al., 2015). These findings bear a clinically relevant content, whose potential might be therapeutically translated into strategies for prevention or/and amelioration of metabolic malprogramming, e.g., in the case of Prader-Willi syndrome (Feigerlová et al., 2008) or in the case of intrauterine and/or neonatal malnutrition (Desai et al., 2005) associated with higher risks of developing hyperphagia and obesity in later life.

In some areas of the nervous system, such as the spinal cord and hypothalamus, ghrelin rather than DAG plays the leading role in adult neurogenesis (Moon et al., 2009b; Inoue et al., 2010). Important to mention here is that in the adult brain fasting increases the density of ARH projections to the PVN, the number of dendritic spines and the density of excitatory synapses (Liu et al., 2012; Cabral et al., 2020). As established by Cabral et al. (2020), this neuronal remodeling is GHSR1a-mediated and depends on the energy balance. Ghrelin’s binding to GHSR1a acutely modulates calcium currents and neurotransmitter release. However, the constitutive activity of the receptor can also impair signaling in chronic, G_*i/o*_- and Ca_V_b-subunit-dependent and voltage-independent manner (López Soto et al., 2015). *In vitro* studies on hippocampal neurons revealed in-depth the role of GHSR1a’s constitutive activity in inhibitory neurotransmission: it leads to a decrease of Ca_V_ channel density at the plasmalemma and a simultaneous increase of their expression at the endoplasmic reticulum and Golgi apparatus (Martínez Damonte et al., 2018). This chronic effect depends on the sustained receptor signaling and not on its subcellular location; it would be more relevant during periods of development, synaptogenesis and adult neurogenesis, when the calcium trafficking is more intensive (Bergami et al., 2015; Nakamura et al., 2015).

Interestingly, ghrelin receptor knock-out fetuses do not display any body weight or food intake abnormalities, possibly owing to a neonatal compensatory mechanism by other developmentally related molecules such as growth factors (Luquet et al., 2005; Sato et al., 2006). Several neurotrophic factors are able to promote neurogenesis in the hippocampus. The stimulatory effect of ghrelin in adult neurogenesis seems to be interceded by the brain derived neurotrophic factor (BDNF) (Bekinschtein et al., 2013). Of note, hippocampal neuronal stem/progenitor cells (NSPCs) do not express GHSR1a (Hornsby et al., 2016) and *in vitro* supplementation of ghrelin to these cells does not lead to proliferation. However, ghrelin increases BDNF mRNA expression in the hippocampus, which explains the stimulatory effect of ghrelin on division and survival of newborn neurons in primary hippocampal cultures, containing both neurons and NSPCs (Hornsby et al., 2020).

**FIGURE 1:**
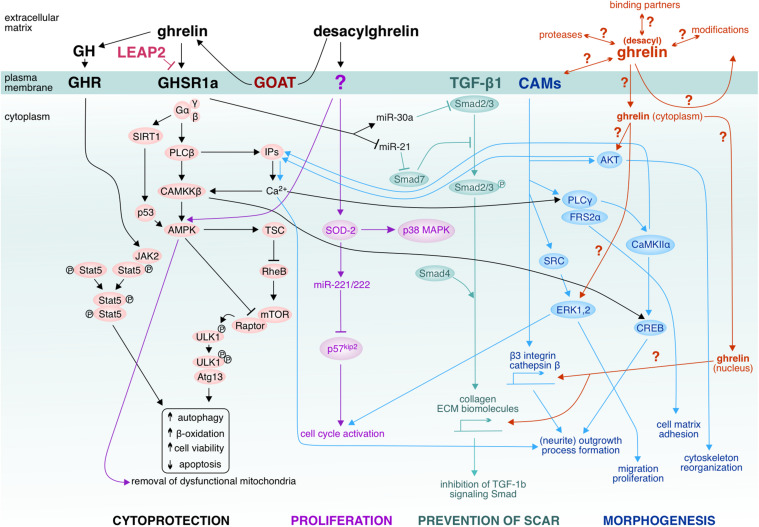
Ghrelin-induced effector pathways and unavowed signaling hotspots. Ghrelin activates the cascade via GHSR1a/CaMKKβ/AMPK to suppress endoplasmic reticulum stress. LEAP2 has been suggested to inhibit GHSR1a. Ghrelin-activated AMPK inhibits mTOR via activation of TSC and inactivation of Raptor, leading to reduced phosphorylation of ULK1 and therefore enhanced ULK1 kinase activity which triggers autophagy (Kim et al., 2011). Ghrelin’s pro-autophagic effect has been shown to improve hepatosteatosis by increasing abundance of mtDNA and inducing mitochondrial free fatty acid β-oxidation. Not only CaMKKβ but also SIRT1-p53 modulates AMPK in the setting of autophagy (e.g., in hypothalamic ghrelin signaling). Ghrelin can act pro-autophagically via the growth hormone (GH) under fat-depleted famine conditions by activating GH receptor-mediated JKA2-pStat cascades. Furthermore, ghrelin has been shown to upregulate antifibrotic (miR-30a) microRNA and downregulate profibrotic (miR-21) microRNA, thus affecting the TGF-β1-Smad pathway and ameliorating skeletal muscle fibrosis upon injury. Plasma membrane-associated GOAT has been proposed to locally convert desacylghrelin to ghrelin. Also desacylghrelin can stimulate the AMPK activity in order to induce autophagy by decreasing reactive oxygen species accumulation and apoptosis, thereby protecting e.g., cardiomyocytes from ischemic injury. Furthermore, desacylghrelin has been shown to stimulate SOD-2 which leads to increased expression of miR-221 and miR-222. In turn, these miRs suppress p57kip2 expression in satellite cells of skeletal muscle, thereby accelerating cell cycle re-entry and proliferation. These events facilitate muscle regeneration. Desacylghrelin-mediated SOD-2 upregulation also increases myogenesis and decreases reactive oxygen species generation, thus promoting tissue regeneration after injury (Yanagi et al., 2018). It has remained unclear whether desacylghrelin/ghrelin can be internalized by the target cells and may act in the cytoplasm, e.g., on AKT or ERK1,2 or by far, whether ghrelin can translocate to the nucleus and affect gene expression. Does ghrelin also associate with cell adhesion molecules (CAMs) or other binding partners at the plasma membrane and/or undergo recycling? It is possible that the desacylghrelin/ghrelin-mediated signaling cascades might be highly intertwined with the signaling cascades mediated by the CAMs. CAMs, such as cadherins, form signaling units with TGF-β1. The interplay between CAMs with their binding partners leads to recruitment of catenins and junction plakoglobin to the nucleus and regulate transcription [e.g., α-catenin can regulate actin bundling (AKT)]. CAMs convey also signals to kinases (for example, SRC family kinases and the Tyr-protein kinase) and phosphatases (PLCγ). Given that the CAM-mediated cascades govern morphogenic events such as cytoskeletal reorganization, process formation, neurite outgrowth and dynamic events like proliferation and migration (Cavallaro and Dejana, 2011), it is conceivable that one possible action of ghrelin to stimulate regeneration could be via affecting these CAM-cascades. AKT (actin); AMPK (5’ adenosine monophosphate-activated protein kinase); Atg13 (autophagy-related); CaMKIIα (Ca^2+^-calmodulin-dependent kinase); CAMKKβ (Calcium/calmodulin-dependent protein kinase kinase 2); CREB (cAMP response element-binding protein); ECM (extracellular matrix); ERK1,2 (extracellular regulated kinase); Frs2α (substrate of fibroblast growth factor receptor); GHSR1a (growth hormone secretagogue receptor 1a); Gα,β,γ (G protein alpha, beta and gamma subunits); IP_3_ (inositol triphosphate); JAK2 (Janus kinase 2); LEAP (Liver-expressed antimicrobial peptide 2); mTOR (mechanistic target of rapamycin); P38 MAPK (p38 mitogen-activated protein kinases); P53 (protein p53); P57^*kip2*^ (Cyclin-dependent kinase inhibitor 1C); PLCβ (phospholipase C); PLCγ (phospholipase); Raptor (Regulatory-associated protein of mTOR); RheB (Ras homolog enriched in brain); SIRT1 (NAD-dependent deacetylase sirtuin-1); Smad2/3, Smad4, Smad7 (Mothers against decapentaplegic homolog 2/3/4/7); SOD-2 (Superoxide dismutase 2); SRC (Src kinase); Stat5 (Signal transducer and activator of transcription 5); TSC (tuberous sclerosis complex); ULK1 (Unc-51 like kinase-1).

The role of ghrelin in adult neurogenesis was also confirmed in ghrelin knock-out mice (Li et al., 2013), GOAT knock-out mice and patients suffering from chronic neurodegenerative disorders (Hornsby et al., 2020). The neurogenic effect of ghrelin is due to activation of multiple signaling pathways, such as extracellular signal-regulated protein kinase 1 and 2 (ERK1/2), phosphoinositide 3-kinase (PI3K), Akt/glycogen synthase kinase (GSK)-3 β, PI3K/Akt/mammalian target of rapamycin (mTOR)/p70^S6K^, and Janus kinase (JAK) 2/signal transducer, and activator of transcription (STAT) 3 signaling pathway (Chung et al., 2013; Figure 1). As a result, ghrelin increases nuclear expression of the transcription factor E2F1 (triggers progression from G_1_ to S phase of cell cycle) and protein levels of positive regulators of the cell cycle cyclin A and cyclin-dependent kinase 2 (CDK2), and at the same time ghrelin downregulates the inhibitors of CDK2, protein p27^KIP1^ and p57^KIP2^ (Chung and Park, 2016). Thus, ghrelin controls the cell cycle of hippocampal neural stem cells bi-directionally: the peptide stimulates the positive and inhibits the negative regulators (see also Figure 1).

The ability of ghrelin to regulate the cell cycle is also relevant for tumor progression (Zhu et al., 2018), especially for hormone-dependent tumor cells (Holly et al., 1999; Jeffery et al., 2003). Many studies report co-localization of ghrelin and GHSR1a and/or GHSR1b in specimens from malignant entities [for a review see Nikolopoulos et al. (2010), Kraus et al. (2016)], thus implicating an autocrine/paracrine role of ghrelin in cell proliferation (Jeffery et al., 2002). However, the functional roles of ghrelin in the modulation of cancer cell fate are controversially debated. A study has reported that ghrelin stimulates oral tumor proliferation through phosphorylation of GSK-3β and nuclear translocation of β-catenin, and up-regulation of the target genes cyclin D1 and c-myc (Kraus et al., 2016). Ghrelin may also contribute to cancer metastasis, since it increases expression, nuclear translocation and promoter-binding activity of Snail, a transcriptional repressor of E-cadherin (Lin et al., 2015). The effect is dose-dependent: low doses promote tumor proliferation, whereas high doses suppress cell growth (Nikolopoulos et al., 2010). It is very unlikely that ghrelin has cancerogenic effects, because the protein acts rather cell protectively by suppressing inflammation, apoptosis, and oxidative stress. But for all that, there is still no clear answer to whether ghrelin may attenuate malignancy and this matter requires further investigation.

The experimental evidence that ghrelin affects neurotrophic factors has led to the question of whether ghrelin gives similar effects as the neurotrophic factors do when being therapeutically applied to injured tissue. In experimental injury models, the protective effects of exogenously administered growth factors depends on the way and time of their administration. For example, intravenous infusion of basic fibroblast growth factor (βFGF) for three consecutive days prior to permanent middle cerebral artery infarction and temporary bilateral carotid artery occlusion reduces the infarct size by 25% in rats (Koketsu et al., 1994; Sugimori et al., 2001). No such morphological effect has been observed when βFGF was injected intracisternally 24 h after stroke, in spite of the achieved functional recovery (Kawamata et al., 1997). Similarly, when ghrelin was used as a protective agent postoperatively (Bianchi et al., 2016b) or in an animal model of liver injury (Mao et al., 2015b), it triggers different molecular mechanisms of repair. For instance, time course analysis of ghrelin-induced anti-fibrotic effect on gene expression showed that ghrelin does not activate fibrinolytic pathway but rather suppresses the initiators of inflammation except for the matrix metalloproteinases: at day 1 post-surgery, ghrelin significantly downregulates transforming growth factor beta 3 (TGFβ3), TGFβ-receptor 2, Interleukin 4 (IL4), IL13 receptor α2 (IL13rα); at day 4 it activates TGFβ3, tumor necrosis factor, IL4, IL13rα2, Smad7; at day 20 – TGFβ2, IL4 (Bianchi et al., 2016b). Ghrelin up-regulates Smad7 at day 4, prevents Smad3 and/or Smad2 phosphorylation and recruitment of Smad2/Smad3 complex which blocks βTGFb activity, as shown in some organs (Nakao et al., 1999; Fukasawa et al., 2004; Dooley et al., 2008)8), and thus, prevents fibrosis (see also Figure 1). Clear conditioning- and time-related patterns of ghrelin’s expression have been detected in neonatal dissociated cultured cortical neurons of rats with high expression levels very early, which decrease as neurons maturate during the next 2 weeks (Stoyanova et al., 2009a,b). This matches the *in vivo* time course of network formation, the survival of which depends on synaptic consolidation and activation (Romijn et al., 1981; Voigt et al., 2005). Such findings suggest that ghrelin influences early synapse formation and neuronal communication, both of which are crucial for brain development and functioning.

**FIGURE 2:**
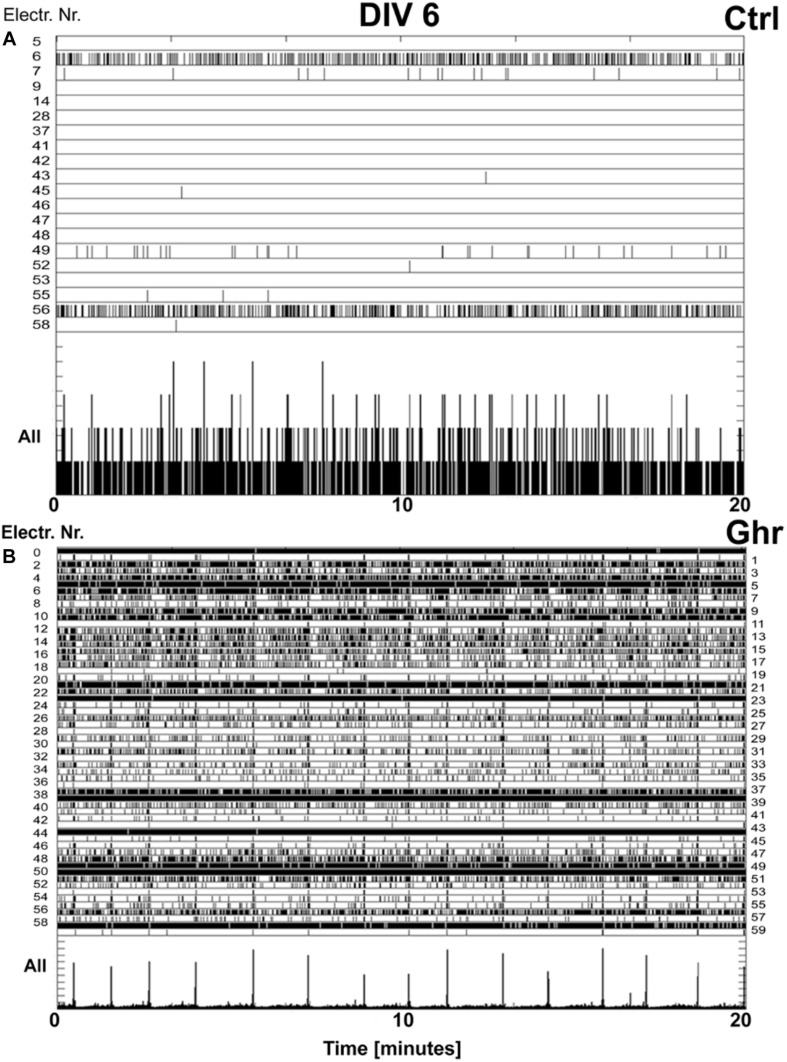
Effect of ghrelin on network activity. Raster plots of the neuronal activity recorded over 20 min (*x*-axis) in cultures incubated with ghrelin and controls at age 6 days *in vitro* (DIV). The top rows of the panels depict only the electrodes in contact with active neurons (electrode numbers are on *y*-axis). Each tick represents a recorded action potential. The bottom rows of the two panels represent the summed network activity. **(A)** Network activity in a sister culture under control conditions, 47 spikes recorded in 1 min, immature activity pattern. **(B)** Network activity in a culture chronically treated with ghrelin, 3,740 spikes recorded in 1 min, showing mature activity pattern. With permission from BMC Neuroscience (Stoyanova and le Feber, 2014).

The morphological substrate of learning and memory consolidation is the neuronal networks. The main phases of their development and activation have been extensively studied in dissociated stimuli-deprived neurons (Kamioka et al., 1996; Chiappalone et al., 2006). The natural evolvement of these circuits includes three stages. Initially, early activity-independent wiring yields an excessive number of synapses. During the second stage, use-dependent pruning of all inappropriate connections occurs (Tessier and Broadie, 2009). Finally, a homeostatic balance between the development of synaptic contacts and intrinsic bioelectrical activity is being established (Corner et al., 2002). Experiments with “brain-on-a-chip” models have also provided a clear electrophysiological evidence that ghrelin has a strong stimulating effect on developing neuronal cultures (see Figure 2): it leads to earlier network activation by accelerating synaptogenesis – a week ahead of the controls (Stoyanova and le Feber, 2014). Remarkably, the cultures chronically treated with ghrelin maintain healthy firing levels higher than those of the controls, possibly due to a predominating formation of excitatory synapses (Stoyanova and le Feber, 2014). The balance between excitation and inhibition is essential for proper neuronal functioning and it is maintained by a form of homeostatic plasticity (Nelson and Turrigiano, 1998; Turrigiano, 2008). By sustaining high activity levels, ghrelin probably enables viability and longevity of the neuronal circuits. This reveals an extraordinary potential of the neuropeptide to counteract low or lacking neuronal activity under pathological conditions such as stroke (Stoyanova and le Feber, 2014).

One could speculate that ghrelin causes neuronal hyper activity leading to epilepsy, supposedly due to excessive network excitability (Chiappalone et al., 2009). However, 30–40% of the epilepsy patients do not respond to treatment that had been aiming at suppressing hyper activity, which suggests that certain forms of epilepsy may have etiology other than hyper excitability. Indeed, it has been shown that hyper excitability could be triggered by insufficient external input, such as *status epilepticus* during sleep (Patry et al., 1971) or deep anesthesia (Gratrix and Enright, 2005). *In vitro* experiments have shown that treatment with mild excitatory agents such as acetylcholine, serotonin, orexin, and ghrelin (Corner, 2008; le Feber et al., 2014; Stoyanova and le Feber, 2014) transforms the neuronal firing patterns into more dispersed and asynchronous ones. On the contrary, strong classical excitatory transmitters like glutamate or aspartate not only do not suppress epileptiform bursting but rather enhance it at specific concentrations (Frega et al., 2012).

In addition to the previously mentioned mechanisms of neuroprotection, ghrelin is able to influence the process of autophagy as shown in various *in vitro* and *in vivo* models of neurodegenerative disorders (Aveleira et al., 2015b; Ezquerro et al., 2017; Akalu et al., 2020). Under normal conditions, autophagy is responsible for removal of excessive cytoplasmic contents and debris, whereas during stress or starvation autophagy could be an alternative energy providing process (Rodríguez et al., 2015). Oxygen-glucose deprivation in neurons entails a considerable autophagy culminating in neuronal damage and death. Ghrelin has been shown to prevent this event chain by inhibiting reactive oxygen species production, stabilizing mitochondrial integrity and transmembrane potential, blocking of cytochrome c release, and inactivating caspase-3-mediated cascades (López-Lluch et al., 2006; Chung et al., 2007, 2018; Akalu et al., 2020). Several other mechanisms of how ghrelin suppresses autophagy have been proposed, e.g., ghrelin can initiate the PI3K/Akt/Bcl-2 signaling (Mao et al., 2015a), which inhibits the glycogen synthase kinase (GSK)-3β and stabilizes β-catenin (Chung et al., 2008). However, aging and variety of neuropathies appear to hinder the process of autophagy (Sinha et al., 2017; Yuan and Wang, 2020), leading to accumulation of damaged cellular constituents and imbalance in cellular homeostasis. On the contrary, in corresponding experimental models ghrelin was found to stimulate neuronal autophagy (Aveleira et al., 2015a). A proposed mechanism involves direct triggering of AMP-activated protein kinase to improve glucose and lipid metabolism (Ezquerro et al., 2017) or via interaction with neuropeptide Y-ergic neurons in the hypothalamus (Andrews, 2011; Müller et al., 2015). Taken together these findings clearly demonstrate two antipodal effects of ghrelin on autophagy and suggest that depending on the conditions, ghrelin plays a neuroprotective role as an “on-demand” autophagy modulator.

### Ghrelin-Mediated Neuroprotection and Repair Upon Acute and Chronic Degeneration

It has been deemed desirable by many researches to test whether ghrelin could stimulate recovery of the brain and spinal cord after aberrant metabolic state and hypoxic/anoxic or mechanical injury. These pathological conditions are manifested, in addition to other processes, by accumulation of microglial cells, which are the first line of defense in the nervous system. Ghrelin has been proven to act as a neuroprotective agent in different animal models of nervous system injury such as cerebral ischemia/reperfusion (Miao et al., 2007), hippocampal neuronal damage (Liu et al., 2006; Xu et al., 2009), degeneration of dopaminergic neurons (Moon et al., 2009a), spinal cord and brain trauma (Bansal et al., 2010; Lee et al., 2010; Besecker et al., 2018). Spinal cord injury is followed by chronic demyelination of the nerve fibers due to apoptotic cell death of oligodendrocytes. The process is triggered by accumulation of microglial cells, which release pro-nerve growth factor (proNGF) and reactive oxygen species (ROS) via activation of p38 mitogen activated protein kinase (p38MAPK) and c-Jun N-terminal kinase (JNK) (Yune et al., 2007; Chorny et al., 2008). As a matter of fact, microglial cells do not express GHSR1a (Moon et al., 2009a), but ghrelin treatment of the injured spinal cord showed significant attenuation of microglial activity, though the exact mechanism was not established in that study (Lee and Yune, 2014). In Alzheimer’s disease or its animal models, microglial cells accumulate at the sites of insoluble fibrillary β-amyloid protein (fAβ) deposition, which binds to their CD36 receptor and thus stimulates pro-inflammatory cytokines secretion (El Khoury et al., 2003). The notion that CD36 receptor also contains a binding sequence for growth hormone secretagogues (Demers et al., 2004) prompted studies seeking molecules able to prevent microglia activation. Interestingly, DAG but not ghrelin counteracts fAβ stimulation of interleukin (IL)-1β and IL-6 mRNA expression in microglial cells (Bulgarelli et al., 2009). However, the authors do not rule out the possibility that this effect could be observed only in the experimental model of cells expressing high levels of transfected receptor.

In ghrelin knock-out and GHSR1 deficient mice a greater loss of dopaminergic neurons in substantia nigra (SN) has been registered when compared to PD model or wild-type animals (Andrews et al., 2009). Along with the findings that postprandial plasma ghrelin levels are lower in PD patients (Unger et al., 2011), the neuroprotective role of ghrelin in PD is characterized by apoptosis suppression, reduction of microglia activity and attenuated local inflammation in SN (Dong et al., 2009; Moon et al., 2009a). Ghrelin targets the AMP-activated protein kinase (AMPK) in dopamine neurons, as recently reported in an *in vitro* study (Bayliss et al., 2016) as well as in different animal models of PD (Horvath et al., 2011; Ng et al., 2012). In contrast, other *in vitro* studies have reported that APMK overactivation leads to α-synuclein accumulation and inhibition of neurite growth (Jiang et al., 2013). Is not yet clear if there is a local ghrelin accumulation or elevated expression at the site of neuronal lesions and this issue requires further experimental evidence.

Growth factors as well as ghrelin activate different signaling cascades under different conditions (Cairns and Finklestein, 2003). The neuroprotective effect of βFGF, at least in part, is probably due to the elevated expression of the antiapoptotic protein bcl-2 (Ay et al., 2001), a mechanism also attributed to ghrelin (Sun et al., 2016). Repeated ghrelin administration on chronically constricted sciatic nerves in rats ameliorates axonal regrowth and myelin repair, and may have antinociceptive effects (Guneli et al., 2010). βFGF also has such a neuroprotective effect, since it activates proliferation, migration and differentiation of neural precursor cells (Palmer et al., 1995). However, treatment of traumatic brain injury (TBI) with ghrelin reduces the level of βFGF and FGF-binding protein (FGF-BP) (Shao et al., 2018). Ghrelin inhibits βFGF-mediated angiogenesis, which suggests that exogenous ghrelin probably competitively inhibits βFGF/FGF-BP stimulated neurovascularisation, and thus, ameliorates the recovery after TBI via another molecular mechanism (Shao et al., 2018). Therefore, it remains unclear whether the relationship between ghrelin and βFGF is causative or competitive – an issue which might need further scientific attention.

Ghrelin (Chung et al., 2007) and its synthetic analog, the GH-releasing peptide-6 hexarelin (Delgado-Rubín de Célix et al., 2006), when applied intracerebroventricularly in a neonatal rat model of unilateral hypoxic-ischemic injury, reduces the injury area in various parts of the brain with a dominating effect in the hippocampus (Brywe et al., 2005). Both molecules stimulate progenitor cell proliferation (Johansson et al., 2008). Ghrelin has been shown to protect cultured primary hypothalamic neurons from stress induced upon oxygen-glucose deprivation as well as to damper neuronal stress in rats after a transient middle cerebral artery occlusion (Chung et al., 2007). This neuroprotective effect has been observed *in vivo*, in a rat model of cerebral ischemia: ghrelin increases the tolerance of hippocampal and cerebral cortex neurons to the ischemic injury by inhibition of apoptosis (Liu et al., 2006; Chung et al., 2008). Ghrelin-treated mice that had been subjected to traumatic brain injury show significant functional recovery due to enhanced preservation of neurons, inhibition of neuronal apoptosis, and prevention of blood–brain barrier breakdown (Lopez et al., 2012).

Ghrelin and GHSR1a agonists suppress the onset of chemically induced epileptic seizures (Obay et al., 2007; Portelli et al., 2012). Interestingly, serum levels of ghrelin decrease rapidly after epileptic attacks and this expression level drop lasts for 24 h, thus awakening researchers’ interest as a diagnostic marker for patients that had suffered a recent epileptic seizure or other paroxysmal events (Aydin et al., 2011). Similar phenomenon has been observed in individuals with chronic neurodegenerative diseases. In patients with Huntington’s disease postprandial ghrelin expression is suppressed and worsens as the locomotor impairment advances, so that the dynamics of the plasma levels of ghrelin could be used for assessment of disease’s progression (Aziz et al., 2010; Weir et al., 2011). In a mouse model of PD, ghrelin protects the nigrostriatal dopamine function by activating mitochondrial uncoupling protein 2 (UCP2)-dependent mechanisms (Andrews et al., 2009). These three neurodegenerative diseases are often accompanied by memory deficits. While in epilepsy memory impairment is due to inhibition of the adult hippocampal neurogenesis (Butler and Zeman, 2008), in ischemic injury it is caused by neuronal loss within the hippocampus (Squire and Zola, 1996).

Memory deficits could be counteracted by ghrelin administration, as supported by several experiments (Carlini et al., 2002, 2008). Intra-amygdaloid injection of ghrelin affects passive avoidance learning and memory, while the intra-hippocampal application improves the long-term memory, but it does not modify the short-term memory. These findings suggest that ghrelin probably modulates specific molecular intermediates involved in memory acquisition/consolidation but not retrieval (Carlini et al., 2010). Moreover, the described effects are ghrelin-specific, because they are eliminated or reversed after treatment with an antagonist of GHSR1a (Tóth et al., 2009, 2010). Systemic application of ghrelin to the hippocampus of ghrelin knock-out mice leads to an increase in spine density in CA1, and thus, improves the performance in cognitive behavior (Diano et al., 2006). Regarding that cognitive impairment may occur in type 2 diabetes patients and that ghrelin also modulates insulin sensitivity (Barazzoni et al., 2008), administration of the peptide to diabetes patients might have a clinical relevance in cognitive behavior recovery (McNay, 2007). Taken together, all these studies provide a link between the role of ghrelin in the metabolic control and the higher brain functions. Moreover, the experimental data suggest that ghrelin has a huge translational therapeutic potential for treatment of learning and memory deficits which are associated with aging and neurological disorders.

## Discussion

Ghrelin expression does not always correlate with GHSR1a expression. It seems that their ratio varies between the different tissues, e.g., in the spinal cord ghrelin could not be detected (Lee et al., 2010), whereas the telencephalon and diencephalon have been found to contain ghrelinergic neurons (Cowley et al., 2003; Hou et al., 2006; Stoyanova, 2012; for further review see Cabral et al., 2017). Motoneurons, oligodendroglia (Lee et al., 2010) and autonomic preganglionic neurons (Furness et al., 2012) are abundantly equipped with GHSR1a. Also the majority of the cultured cortical neurons are positive for GHSR1a (Stoyanova and le Feber, 2014). Thus, every neural cell carrying GHSR1a could be a potential target of ghrelin. The wide distribution of the receptor allows the neuropeptide to influence multiple regions of the nervous system and it makes the systemic ghrelin application convenient when neuroprotection is needed for treatment of the diseased or injured nervous system.

One has but to consider that systemic administration of ghrelin cannot be expected to provide the desired therapeutic concentration at the site of injury, because the circulating esterases might deacylate it (De Vriese et al., 2004), thus preventing sufficient amount of ghrelin to activate GHSR1a. Therefore, rather a local administration of ghrelin might be far more effective than the systemic or intraventricular ones. On the other hand, the enzymes [e.g., butyrylcholinesterases and acetylcholinesterases in the meninges (Ummenhofer et al., 1998)] present at the place of ghrelin application can deacylate it, thus urging the co-application of ghrelin together with enzyme-inhibitors. Inhibition of proinflammatory cytokines and/or of LEAP2 would allow ghrelin to access its receptor and unfold its neuroprotective functions. Indeed, a ghrelin mimetic alone fails to prevent hippocampal lesions in a mouse model of Alzheimer’s disease pathology, although it improves neurogenesis (Tian et al., 2019).

Dependent on the type and complexity of injury, the local administration of ghrelin cannot be always suitable, e.g., in the case of autoimmune encephalomyelitis. The latter is characterized by a systemic loss of oligodendroglia, demyelination, and inflammation, all of which could be prevented by subcutaneous injection of ghrelin to an animal model of the disease (Tian et al., 2019). Nevertheless, both types of application of ghrelin (local and systemic) have yielded similar outcome. The neuroprotective effect of ghrelin is robust and reproducible as successfully demonstrated in a variety of animal models of neurodegenerative diseases and other neuronal injuries such as ischemia, traumatic brain injury, spinal cord injury, and amyotrophic lateral sclerosis (for a review, see Fields et al., 2011; Dos Santos et al., 2013; Stoyanova, 2014). Still, the concept of the therapeutic ghrelin/GHRS1a ratio and the way of administration requires further experimental attention.

Following this notion, it appears indispensable to determine the effective dose of ghrelin facilitating neuroprotection; does such a dose match the dose triggering eating behavior? The plasma concentration of ghrelin after the consumption of fat-rich meat has been found to reach ∼500 pg/ml (Erdmann et al., 2004). This corresponds to 0.1483 μM. Compared with the pre-prandial concentration of ghrelin (0.117 μM), the post-prandial increase of the peptide level is 1.3-fold. Studies on the synaptogenic and neuroprotective effects of ghrelin have revealed that the administered doses of ghrelin may vary from 0.3- to 3.4-fold of the average physiological pre-prandial ghrelin plasma concentration (Diano et al., 2006; Lee et al., 2010; Stoyanova et al., 2013). The fact that Diano and colleagues have recorded synaptogenic effects upon administration of 10 μg/kg ghrelin, which is 0.3-fold of the pre-prandial levels, suggests that ghrelin acts neuroprotectively at doses much lower than those initiating feeding. This would have one benefit regarding the neurodegenerative diseases where ghrelin levels are decreased: even under low energy conditions ghrelin’s neuroprotective effect can still be maintained.

It should be noted that nervous system injury does not only include damage of neurons but also alteration of the extracellular space. So far, the ghrelin research field has focused on finding therapeutic approaches targeting intracellular repair mechanisms. However, the interstitial changes at and around the lesion site should not be neglected, as they are related to disintegration of the extracellular matrix, damaged vascularization, and metabolic spill-out. Extracellular proteins and matrix proteases, cell adhesion molecules, neurotrophic factors as well as different organic and inorganic compounds of the nervous tissue are necessary for recovery after injury. For example, serine proteases which deacylate ghrelin cleave also neuronal adhesion molecules and facilitate variety of morphogenic and dynamic events such as neurite outgrowth, process formation, myelination, synaptogenesis, cell migration, and extracellular matrix reorganization. It has still remained unclear how ghrelin could harness all these molecules in the healing process. Few studies on non-neural tissues have shown that ghrelin inhibits the expression of transforming growth factor β1 (TGF-β1) and phospho-Smad3 (Mao et al., 2015b; Sun et al., 2015). Ghrelin also suppresses collagen production (Ota et al., 2013), upregulates antifibrotic microRNA and downregulates profibrotic microRNAs in skeletal muscle after injury, leading to inactivation of the TGF-β1/Smad pathway and alleviation of fibrosis (Katare et al., 2016). Studies in both humans and mouse (Moreno et al., 2010; Bianchi et al., 2016a,b) allowed defining in depth the activated cascades (see also Figure 1).

Since recovery recapitulates ontogenetic processes, where ghrelin plays a significant role, one could conclude that the neuropeptide would stimulate developmentally related molecules, neurotrophic and transcription factors if applied therapeutically to the injured tissue. Regarding the very low physical concentration of DAG/ghrelin in the central nervous system, it is not clear how endogenous DAG/ghrelin could be provided to the lesion site or be increased locally to stimulate regeneration. One possible way of intrinsic supply would be by binding to bystander proteins on the surface of blood cells or cells of the immune system, which penetrate the injured tissue owing to ruptures in the blood-brain barrier. Also in the process of endothelial mesenchymal transition the newly formed blood vessels within the injured tissue as well as the surrounding areas may supply ghrelin. Locally, the continuously supplied DAG may then be converted to ghrelin by the plasma membrane-associated GOAT. It can be also hypothesized that DAG/ghrelin may interact with CAMs/TGFβ1 to facilitate neuroprotection (Figure 1). It is also not clear whether DAG/ghrelin is internalized by neuronal cells. One cannot exclude that ghrelin may act cytoplasmatically and that this 28 amino acid-long peptide might translocate into the nucleus to modulate the expression of genes modulating remodeling and repair (Figure 1). Such in-depth studies on the injured nervous system still need to be carried out.

Undisputedly, ghrelin is an omnipotent molecule, which puts the organism in the condition of recovery. However, careful analyses of the interspecies differences are needed in order to evaluate to what extent we could extrapolate our results from animal models to humans.

## Author Contributions

IS and DL carried out the literature survey and wrote the manuscript. Both authors contributed to the article and approved the submitted version.

## Conflict of Interest

The authors declare that the research was conducted in the absence of any commercial or financial relationships that could be construed as a potential conflict of interest.

## Footnotes

1 https://pubchem.ncbi.nlm.nih.gov/compound/
